# Mitochondrial Dysfunction as a Potential Mechanism Mediating Cardiac Comorbidities in Parkinson’s Disease

**DOI:** 10.3390/ijms252010973

**Published:** 2024-10-12

**Authors:** Agustina Salis Torres, Ji-Eun Lee, Andrea Caporali, Robert K. Semple, Mathew H. Horrocks, Vicky E. MacRae

**Affiliations:** 1 The Roslin Institute and R(D)SVS, University of Edinburgh, Easter Bush, Midlothian EH25 9RH, UK; asalist@ed.ac.uk (A.S.T.); jlee20@ed.ac.uk (J.-E.L.); 2 EaStCHEM School of Chemistry, University of Edinburgh, Edinburgh EH9 3FJ, UK; mathew.horrocks@ed.ac.uk; 3 IRR Chemistry Hub, Institute for Regeneration and Repair, University of Edinburgh, Edinburgh EH16 4UU, UK; 4 Centre for Cardiovascular Science, Queen’s Medical Research Institute (QMRI), The University of Edinburgh, 47 Little France Crescent, Edinburgh EH16 4TJ, UK; acaporal@exseed.ed.ac.uk (A.C.); rsemple@exseed.ed.ac.uk (R.K.S.); 5 MRC Human Genetics Unit, Institute for Genetics and Molecular Medicine, The University of Edinburgh, Edinburgh EH4 2XU, UK

**Keywords:** mitochondria, mitochondrial dysfunction, Parkinson’s disease (PD), cardiac dysfunction

## Abstract

Individuals diagnosed with Parkinson’s disease (PD) often exhibit heightened susceptibility to cardiac dysfunction, reflecting a complex interaction between these conditions. The involvement of mitochondrial dysfunction in the development and progression of cardiac dysfunction and PD suggests a plausible commonality in some aspects of their molecular pathogenesis, potentially contributing to the prevalence of cardiac issues in PD. Mitochondria, crucial organelles responsible for energy production and cellular regulation, play important roles in tissues with high energetic demands, such as neurons and cardiac cells. Mitochondrial dysfunction can occur in different and non-mutually exclusive ways; however, some mechanisms include alterations in mitochondrial dynamics, compromised bioenergetics, biogenesis deficits, oxidative stress, impaired mitophagy, and disrupted calcium balance. It is plausible that these factors contribute to the increased prevalence of cardiac dysfunction in PD, suggesting mitochondrial health as a potential target for therapeutic intervention. This review provides an overview of the physiological mechanisms underlying mitochondrial quality control systems. It summarises the diverse roles of mitochondria in brain and heart function, highlighting shared pathways potentially exhibiting dysfunction and driving cardiac comorbidities in PD. By highlighting strategies to mitigate dysfunction associated with mitochondrial impairment in cardiac and neural tissues, our review aims to provide new perspectives on therapeutic approaches.

## 1. Introduction

Parkinson’s disease (PD) is the second most common neurodegenerative disorder, impacting 1% to 3% of individuals aged over 65. With longer life expectancies, both the prevalence and impact of PD are growing globally [1]. Advanced aging is also related with the increasing prevalence of cardiac dysfunction and, therefore, it is reasonable to explore potential connections between PD and cardiac issues [2]. Individuals with PD exhibit a heightened susceptibility to cardiac dysfunction, suggesting a complex interplay between the two conditions [1,3,4,5,6,7]. Cardiovascular disorders have been identified in approximately 80% of PD patients [8]. Several studies have highlighted the association between PD and increased risks of myocardial infarction, congestive failure and all-cause mortality in PD patients [1,9]. A case-control study with a three-year follow-up observed a significantly increased risk of acute myocardial infarction in PD patients [9]. Additionally, PD patients may develop cardiomyopathies, arrhythmias, or sudden cardiac death, though the incidence is debated [10]. The mechanisms mediating these associations, whether direct or indirect, remain unproven. However, aberrant protein aggregation, clearance disruption, oxidative stress, neuroinflammation, and mitochondrial damage have all been suggested as potential contributors [1]. Notably, mitochondrial dysfunction and shared oxidative mechanisms have been proposed as candidate factors mediating the heightened risk of cardiac dysfunction in PD [3].

Given the extensive evidence of mitochondrial dysfunction in the development and progression of both cardiac dysfunction and PD, it is plausible that these two conditions share common underlying pathophysiological mechanisms, thereby potentially mediating cardiac comorbidities in PD patients. Mitochondria serve as dynamic cellular powerhouses, centrally involved in adenosine triphosphate (ATP) generation through oxidative phosphorylation (OXPHOS) [11]. This functionality impacts essential cellular processes, such as metabolism, redox balance, calcium (Ca^2+^) regulation, apoptosis and signal transduction, forming an interconnected network [12]. Compromised mitochondria lead to reduced energy production, altered cellular metabolism and disrupted regulation of reactive oxygen species (ROS). These changes collectively contribute to cellular damage and dysfunction, initiating a pathophysiological cascade associated with both cardiac dysfunction and PD [13,14,15]. Recognising this overlap suggests a promising avenue for integrated treatment strategies targeting the common underlying mechanisms of both conditions. By exploring these shared pathways, this review aims to address research gaps and propose integrated therapeutic strategies focusing on mitochondrial dysfunction to manage both cardiac comorbidities in PD patients as well as the management of PD itself, emphasising the need for future investigations.

### 1.1. Parkinson’s Disease

Parkinson’s disease (PD) is a prevalent neurodegenerative disorder affecting both motor and cognitive functions [16]. Clinical features of PD include bradykinesia, resting tremor, rigidity and later-stage postural instability [17]. Influenced by age, PD prevalence ranges from 0.5–1% in individuals aged 59–69 to 1–3% in those over 80. It is anticipated that by 2030, there will be an increase of over 30% in PD prevalence due to demographic aging [17]. The intricate interplay of genetic and environmental factors contributes to PD development, with 10–15% of cases exhibiting familial links and approximately 5% showing Mendelian inheritance patterns [18]. Polygenic risk factors contribute to the disorder, with 23 ‘PARK’ genes identified [17]. Well-established PD genes linked to mitochondrial dysfunction include autosomal dominant forms (e.g., synuclein-alpha (SNCA) and leucine-rich repeat kinase 2 (LRRK2) mutations) and autosomal recessive forms (e.g., Parkin RBR E3 Ubiquitin Protein Ligase (PRKN), PTEN-induced kinase 1 (PINK1) and ATPase cation Transporting 13A2 (ATP13A2) mutations) [17,18,19]. Environmental factors thought to influence the development of PD include smoking, caffeine consumption and pesticide exposure [20,21,22]. PD’s primary cause is the loss of dopaminergic (DA) neurons in the substantia nigra pars compacta (SNpc), leading to motor symptoms and reduced dopamine levels [23]. The complexity of PD arises from mechanisms including protein misfolding, aggregation, mitochondrial dysfunction, oxidative stress, energy depletion, excitotoxicity and neuroinflammation [24,25,26,27]. Among these, protein misfolding and accumulation within intracellular spaces stand out as prominent hypotheses in PD aetiology [28]. PD exhibits the presence of misfolded amyloid proteins within the intracellular spaces of SNpc neurons, forming structures known as Lewy bodies. These Lewy bodies encapsulate multiple misfolded amyloid proteins, including α-synuclein, phosphorylated tau (p-tau) and amyloid beta protein (Aβ) [28].

**Figure 1:**
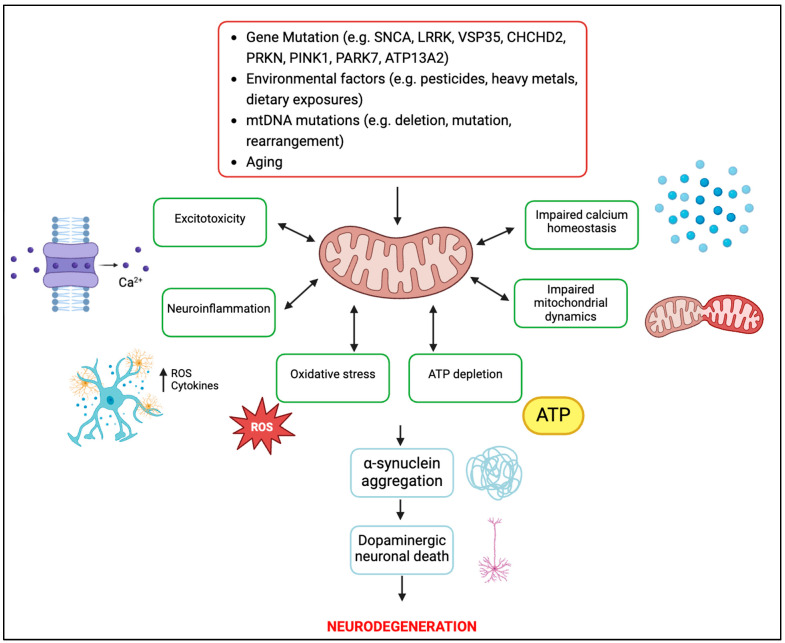
Mitochondrial Dysfunction in Parkinson’s Disease (PD). Various factors, including genetic susceptibility, environmental influences, mtDNA mutations and aging, have been implicated in the onset of Parkinson’s disease. Notably, abnormalities in mitochondrial metabolic function, morphology and homeostasis are observed, which contribute to the formation of α-synuclein aggregates and the subsequent death of dopaminergic (DA) neurons, thereby driving the progression of neurodegeneration. SNCA, Synuclein Alpha; LRRK, leucine-rich repeat kinase 2; VSP35, Vacuolar protein sorting ortholog 35; CHCHD2, Coiled-coil-helix-coiled-coil-helix domain containing 2; PINK1, PTEN-induced kinase 1; PARK7, Parkinson disease protein 7; PRKN, parkin RBR E3 ubiquitin protein ligase; ATP13A2, ATPase Cation Transporting 13A2; mtDNA, mitochondrial DNA; Ca^2+^, calcium ions; ROS, Reactive Oxygen Species; ATP, Adenosine triphosphate.

Mitochondria’s dynamic properties are crucial in neurons due to their substantial energy demands [29]. Mitochondrial dysfunction significantly contributes to PD progression, resulting in heightened oxidative stress and influencing various cellular pathways (Figure 1) [25]. In pathology, α-synuclein undergoes oligomerisation at mitochondrial membranes [30]. These α-synuclein oligomers disrupt the normal functioning of ATP synthase, leading to mitochondrial dysfunction characterised by impaired respiration and the opening of the permeability transition pore (PTP), ultimately leading to neuronal toxicity and cell death [31,32]. This dysfunction further impairs mitophagy, compromises trafficking, alters mitochondrial dynamics and disrupts calcium ion (Ca^2+^) balance [33]. The interaction of α-synuclein oligomers with neuronal membranes disrupts calcium signalling through increased lipid permeability and induction of ion channel formation [34,35]. Therefore, addressing mitochondrial dysfunction in PD presents an attractive avenue for therapeutic interventions in PD management.

### 1.2. Cardiac Dysfunction

Cardiac dysfunction is a major global health concern significantly contributing to morbidity and mortality, particularly in the context of advanced aging. Despite significant progress in the prevention and treatment of cardiovascular diseases (CVD), they remain the leading cause of death worldwide, accounting for approximately 17.9 million deaths in 2019 [36]. The global incidence of these diseases has nearly doubled between 1993 and 2019, with projections indicating a continued rise until 2024 [37]. A significant portion of cardiac dysfunction cases is attributed to coronary heart disease (CHD). CHD involves the narrowing or blockage of coronary arteries due to plaque accumulation, leading to reduced blood flow and contributing to conditions such as angina, myocardial infarction, and heart failure [38]. Myocardial infarction, or heart attack, occurs when blood flow to a part of the heart muscle is severely reduced or completely blocked, causing tissue damage or death due to lack of oxygen [39]. Heart failure is characterised by the heart’s inability to pump blood efficiently, resulting in inadequate circulation to meet the body’s needs [40]. Despite advancements in treatment and management strategies, these conditions continue to pose significant health challenges globally.

The role of mitochondria in cardiac function is gaining attention as a potential therapeutic avenue due to its involvement in several cardiac-related pathogenesis. Established risk factors such as hypertension, insulin resistance, diabetes and obesity have been associated with disruptions in cardiac mitochondrial dynamics, mitophagy irregularities and mitochondrial metabolic dysfunction [41,42,43,44,45,46,47,48,49]. These are also established risk factors in PD [50,51,52,53]. Studies highlight the involvement of cardiac myocardial mitochondrial imbalances in cardiac dysfunction [54,55]. Additionally, aberrant mitochondrial morphology and dysfunction are recognised as significant indicators of heart failure [56]. This highlights a potential role of mitochondria in cellular processes related to the progression of cardiac dysfunction and PD.

## 2. The Many Faces of Mitochondrial Function

**Figure 2:**
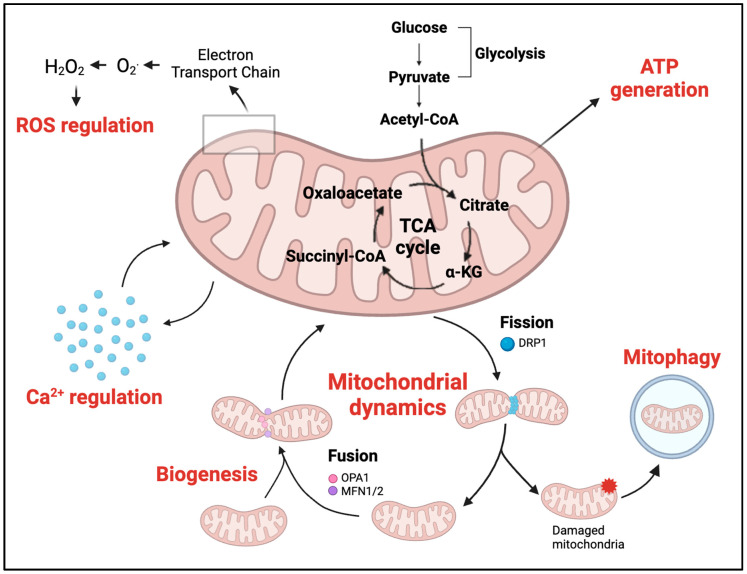
Mitochondrial function. Mitochondria are dynamic organelles crucial for energy generation, highlighted through oxidative phosphorylation. Their functionality is regulated by dynamic processes including fusion, enabling exchange of contents between mitochondria, and fission, facilitating organelle division and distribution. Mitochondrial biogenesis ensures the replenishment of these organelles, balancing turnover and maintenance. Mitophagy removes dysfunctional mitochondria, safeguarding cellular integrity. Regulation of ROS production within mitochondria is essential for redox homeostasis and physiological processes such as cell differentiation, senescence, signal transduction and adaptation to hypoxic conditions. Calcium homeostasis modulates signalling pathways and influences cellular functions. Ca^2+^, calcium ion; ATP, Adenosine triphosphate; DRP1, Dynamin-related Protein 1; OPA1, optic Atrophy 1; MFN1/2, Mitofusin-1/2; H_2_O_2_, hydrogen peroxide; O_2_, dioxygen; TCA, tricarboxylic acid; α-KG, alpha-ketoglutarate; CoA, coenzyme A.

Mitochondria are dynamic cellular structures with a central role in cellular function, primarily serving as the major generators of the chemical energy necessary to fuel the cell’s biochemical reactions [57]. However, their functions extend beyond energy production, encompassing areas such as cellular metabolism, calcium ion (Ca^2+^) regulation, redox signalling, cellular homeostasis and adaptation to stress (Figure 2) [58,59,60]. Their multifaceted functions make mitochondria indispensable components within tissues like the brain and heart, where they play essential roles in maintaining physiological integrity. Both organs, characterised by high metabolic demands, depend on a consistent supply of nutrients to support their functions. The heart maintains a continuous rhythmic beating, and a subset of neurons in the brain remains active at all times, preventing these tissues from entering a state of complete rest [61,62,63]. Additionally, they both undergo rapid shifts in function, as cardiac workload can vary significantly during physical exercise, and individual neurons undergo swift alterations in action potential during specific cognitive tasks [61,64,65]. As a result, mitochondrial dysfunction in these tissues can lead to significant consequences, contributing to the development of neurodegenerative and heart diseases. Here we explore the various roles mitochondria play in both the brain and heart.

### 2.1. The Role of Mitochondria in Brain Function

Despite constituting just 2% of the body’s weight, the human brain exerts a substantial energy demand, accounting for 20% of the body’s total energy consumption [66,67]. The heightened metabolic expenditure, essential for fundamental neural functions within the central nervous system, is primarily attributed to the intricate morphology of neurons, finely regulated ion gradients and ceaseless activity across billions of synapses [68]. Mitochondrial ATP production is essential for various synaptic processes, including the operation of ion channels, pumps, receptors and the release of neurotransmitters through Ca^2+^-induced fusion of synaptic vesicles with the presynaptic membrane (exocytosis), as well as the subsequent recycling of neurotransmitters [69,70,71]. During an action potential, axonal terminals expend a significant amount of ATP—approximately 4.5 × 10^8^ ATP molecules, compared to 3 × 10^6^ ATP for resting potentials and basic cellular maintenance [72]. The majority of neuronal ATP is consumed by membrane pumps like the sodium-potassium pump (Na^+^/K^+^ ATPase) and Ca^2+^ ATPase, accounting for 55% of total production to maintain the resting potential [73]. Synaptic vesicle recycling also demands substantial energy, involving ATP for both glutamate vesicle events and restoring ionic gradient within neuronal terminals [67,74]. Most of the ATP in these cells is primarily generated through oxygen-dependent metabolism within the mitochondria, meeting the brain’s energy demand through the oxidation of glucose via glycolysis and OXPHOS to produce ATP [61,70,71]. In cases of PD, a systemic deficiency in complex I of the ETC leads to ATP depletion, a characteristic observed in many patients with the disease [23]. This was first discovered in the early 1980s when individuals injected themselves with 1-methyl-4-phenyl-1,2,3,6-tetrahydropyridine (MPTP), leading to the accumulation of its toxic metabolite, 1-methyl-4-phenylpyridinium (MPP^+^), in the mitochondria, ultimately causing DA neuronal death by reducing ATP synthesis and increasing ROS production [23,75].

Beyond ATP synthesis, mitochondria also play a critical role in ROS regulation and Ca^2+^ signalling, requiring their mutual interplay for fine-tuning modulation [76]. ROS play a crucial role in both the induction of long-term potentiation (LTP) through redox modifications of target proteins and the regulation of synaptic plasticity-related signalling molecules, receptors and channels [77,78]. For example, ROS bidirectionally modulates Ca^2+^ handling, including cytoplasmic influx, buffering and release from intracellular stores [79]. The precise regulation of ROS production is crucial for normal neuronal function, as conditions that induce oxidative/nitrosative stress can lead to excessive Ca^2+^ release, potentially triggering pathological responses and neuronal cell death, which has been shown to be a significant contributor to DA neuronal loss in PD [80]. In terms of Ca^2+^ regulation, neuronal mitochondria are essential for maintaining intracellular Ca^2+^ homeostasis, either by directly buffering Ca^2+^ or by providing the ATP necessary for the activity of the plasma membrane Ca^2+^ ATPase, effectively modulating cytosolic Ca^2+^ responses during neuronal excitation [69,81]. As a result, mitochondria possess a remarkable capacity to influence numerous Ca^2+^-dependent functions within neurons [81]. In PD, DA neurons in the substantia nigra are susceptible to Ca^2+^-overload via essential voltage-gated L-type channels that regulate their pace making activity [82]. This in turn leads to an increase in oxidative stress, further damaging DA neurons and contributing to the progression of PD [83]. Other factors, such as increased mitophagy, increased mitochondrial DNA (mtDNA) damage and defective mitochondrial biogenesis/dynamics, further contribute to the progression of PD [75].

### 2.2. The Role of Mitochondria in Cardiac Function

**Figure 3:**
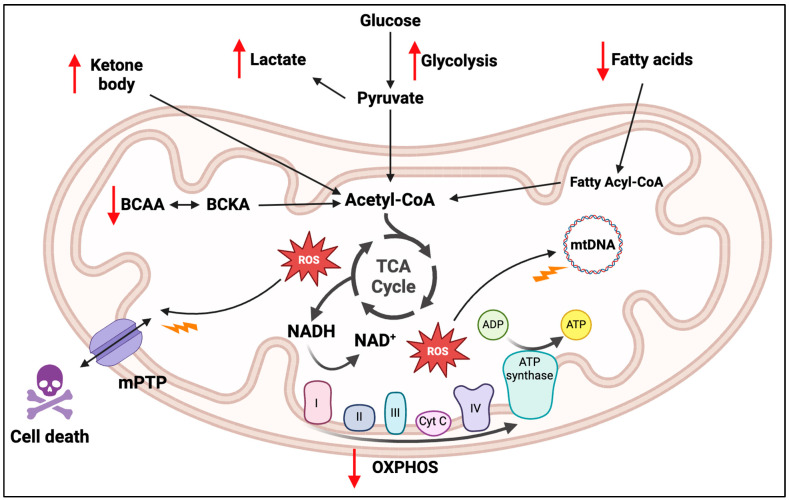
Cardiac metabolism in failing heart. In heart failure, metabolic shifts include decreased fatty acid oxidation, enhanced glycolysis, reduced glucose oxidation, increased lactate, increased ketone oxidation, decreased branched-chain amino acid oxidation and decreased oxidative phosphorylation. The abnormal substrate utilisation results in an increased production of radical oxygen species which can damage mitochondrial DNA and induce cell death by triggering the opening of the mitochondrial permeability transition pore. BCAA, branched-chain amino acids; BCKA, branched-chain keto acid; OXPHOS, oxidative phosphorylation; mPTP, mitochondrial permeability transition pore; NADH, nicotinamide adenine dinucleotide, NAD^+^, Nicotinamide adenine dinucleotide; CoA, coenzyme A; ATP, Adenosine triphosphate; ADP, Adenosine diphosphate; Cyt C, Cytochrome C.

In the heart, mitochondria play a crucial role in meeting the high-energy demands of cardiomyocytes, the primary cell type found in the heart responsible for generating contractile force [84,85]. Mitochondria occupy roughly one-third of the volume within these cells [86]. Moreover, they are responsible for generating over 95% of the ATP in adult cardiomyocytes, which is crucial for facilitating muscle contraction and ion transport [87,88]. The continuous and rapid synthesis of ATP is crucial to sustain this heightened energy flux. In failing human myocardium and in the heart of animal models of severe heart failure, myocardial ATP levels are 30% lower than in healthy myocardium [89,90]. Remarkably, there is a significant correlation between the ATP content in myocardial tissue and in vivo contractile function in human biopsies of failing hearts [91]. Under normal conditions, mitochondria efficiently convert different substrates, including fatty acids, glucose and ketone bodies, into ATP (Figure 3) [92]. Fatty acids are the preferred substrate, accounting for 60–90% of the myocardium’s energy supply [92]. They are metabolised through fatty acid β-oxidation (FAO) within the mitochondria, leading to the generation of acetyl-CoA, which enters the tricarboxylic acid (TCA) cycle, subsequently producing FADH2 and NADH [87]. These electron carriers are crucial for fuelling the electron transport chain (ETC), facilitating ATP synthesis and consuming oxygen [87]. Under conditions of reduced FAO, such as during stress, glucose becomes the primary energy source instead [93]. Glucose undergoes glycolysis in the cytosol, resulting in the production of pyruvate, which is then converted to acetyl-CoA, subsequently entering the TCA cycle and ETC [87]. In cases of heart failure, there is a shift in the utilisation of these mitochondrial energy substrates [94]. Research has shown that hypertrophied hearts have lower rates of fatty acid oxidation and higher rates of glycolysis compared to healthy hearts, indicating a gradual shift in substrate utilisation from fatty acid to glucose consumption [95].

Beyond ATP production, mitochondria also play important roles in regulating Ca^2+^ homeostasis, redox status and lipid synthesis [87]. Particularly, mitochondrial Ca^2+^ plays a pivotal role in regulating cardiac excitation–contraction coupling (ECC) [96]. This regulatory function encompasses two primary aspects: firstly, mitochondrial Ca^2+^ is involved in shaping cytosolic Ca^2+^ signals by effectively absorbing and buffering Ca^2+^ ions, contributing to the fine-tuning of contractile activity within cardiac myocytes [96]. Secondly, mitochondrial Ca^2+^ uptake influences cellular metabolism and the necessary energy supply for muscle contractions, facilitated through a calcium-dependent mechanism extending to key enzymes within the TCA cycle, as well as various components of the ETC and the mitochondrial F1/F0 ATP synthase [96]. In cases of heart failure, energy deficiency becomes intertwined with dysregulated Ca^2+^ transport, resulting in excitation–contraction uncoupling and impaired cardiac function [94]. Redox signalling also holds substantial significance within cardiac ECC. The oxidative modification of specific proteins orchestrates various aspects of the cardiac ECC machinery, influencing processes such as sarcoplasmic reticulum (SR) Ca^2+^ release and uptake, ionic fluxes and contractile function [97]. Additionally, redox signalling extends its influence to other critical physiological processes, including cell differentiation and the maintenance of homeostatic and stress response pathways, such as adaptation to hypoxia/ischemia [98]. In pathological conditions, an excessive accumulation of mitochondrial reactive oxygen species (mROS) can lead to unfavourable cardiac remodelling and fibrosis [98]. Research suggests an excessive amount of mROS to promote sudden cardiac death and cause proteome remodelling [99]. Moreover, the generation of mROS during the early stages of reperfusion has been implicated in the promotion of myocardial ischemia/reperfusion (I/R) injury [94]. In essence, mitochondria hold a critical role in maintaining overall health, and any dysregulation in these cellular structures can significantly contribute to the development of heart failure.

## 3. Mitochondrial Dysfunction

### 3.1. Bioenergetic Deficits

Mitochondrial ATP production is the main energy source for intracellular metabolic pathways [100]. This process involves oxidative phosphorylation (OXPHOS), which comprises two closely interlinked components: (1) the electron transport chain (ETC), located within the inner mitochondrial membrane, and (2) chemiosmosis coupling, driven by an electrochemical gradient [101]. During OXPHOS, electrons derived from substrates like glucose and fatty acids traverse the ETC, simultaneously pumping protons across the mitochondrial membrane. Protons subsequently flow back through ATP synthase, converting adenosine diphosphate (ADP) and inorganic phosphate (Pi) into ATP [102].

Bioenergetic disruptions, primarily associated with mitochondrial dysfunction, are well-documented in cardiac pathophysiology and conditions like PD (Table 1) [62,103,104]. This imbalance in mitochondrial bioenergetics can result in impaired basic functions, affecting oxygen and substrate availability, thus altering metabolism and causing inefficient energy utilisation [103,105]. For instance, during ischemia, compromised OXPHOS and a shift to anaerobic glycolysis lead to reduced intracellular ATP levels, altered pH and lactic acid accumulation [106]. In hypertrophic and failing hearts, initial pressure overload induces a shift from fatty acid to glucose oxidation. This shift leads to a significant decrease in ATP concentration and alterations in substrate utilisation, contributing to a progressive myocardial degeneration [105,106].

Altered metabolism and energy utilisation is also a hallmark in PD. Evidence shows that neurons adopt glycolysis as a low-efficient compensatory mode of energy production in response to mitochondrial dysfunction [103]. A Drosophila PD model based on the inactivation of the DJ-1beta gene (ortholog of human PARK7), showed protein metabolism alterations and a shift from the TCA to glycolytic pathway to obtain ATP, together with an increase in the expression of some urea cycle enzymes [107]. Furthermore, during stress, astrocytes increase lactate production through glycolysis to support neurons. This bioenergetic shift between lactate and glucose levels can alter mitochondrial function and redox pairs that control bioenergetic-pathway feedback, potentially leading to further mitochondrial damage, glutamate toxicity and eventual neurodegeneration [108]. Studies also suggest that increased microglial activation, which display metabolic shifts from oxidative phosphorylation to glycolysis, contributes to amyloid deposition in PD [109,110].

Damage of mitochondrial electron transport chain (ETC) affecting complex I (NADH:ubiquinone oxidoreductase), and a reduction of maximal OXPHOS capacity have been widely reported in cardiac and PD pathogenesis [103,106]. This dysfunction in complex I translates into an increased ROS production and a decrease in energy supply, contributing to disease progression [106,111]. Reduced complex I activity has been identified in the SNpc of PD patients and human failing myocardium [106,112,113,114]. Recent research utilising a mouse knockout model demonstrated that complex I disruption can induce PD [108]. Similarly, defects in single subunits of mitochondrial complex I have been associated with cardiac hypertrophy, ischemia/reperfusion (I/R) injury, as well as diabetic complications and stroke in pre-clinical studies [115]. Mitochondrial bioenergetic deficiencies in the failing heart not only diminish ATP production but also impede high-energy phosphotransferase system activity, including creatine kinase and adenylate kinase [116,117]. This impedes the efficient transfer of ATP to myofibrils, compromising heart contractility. Additionally, reduced myofibrillar ATPase activity leads to decreased energy utilisation efficiency [116].

Antioxidants have become increasingly recognised as promising therapeutic avenues to counter bioenergetic deficits. Coenzyme Q10, an essential antioxidant and integral component of the ETC known to be depleted in PD, has shown promise in reversing complex I deficits observed in PD patient-derived cultures [106,118,119]. Additionally, in preclinical PD models, it demonstrated efficacy in mitigating the loss of dopamine neurons [120]. Similarly, in a canine model of isoproterenol-induced heart failure, coenzyme Q10 supplementation improves left ventricular function [121]. Alpha-lipoic acid (ALA) has also emerged as a powerful antioxidant and cofactor for mitochondrial enzymes involved in energy production. ALA alleviates motor deficits in PD models by regulating iron metabolism, reducing ROS accumulation and protecting mitochondria, ultimately preventing ferroptosis, a type of iron-driven cell death, through the silent information regulator 1/nuclear factor erythroid 2-related factor 2 (SIRT1/NRF2) signalling pathway [122]. Furthermore, ALA exhibits potent antioxidant properties and has potential protective effects against risk factors associated with cardiac dysfunction, including modulation of blood lipids, protection against low-density lipoprotein (LDL) oxidation and regulation of hypertension [123]. This convergence highlights potential mitochondrial-level interplay and suggests shared underlying mechanisms or vulnerabilities related to mitochondrial bioenergetic deficits in both conditions.

**Table 1:** Summary of mitochondrial pathways disrupted in cardiac dysfunction and Parkinson’s disease, and potential common mechanisms.

Pathway	Cardiac Dysfunction	Parkinson’s Disease	Common Mechanisms	References
Bioenergetic Pathways	Impaired OXPHOS leads to reduced ATP production, affecting cardiac contractility. Ischemia shifts metabolism from fatty acid oxidation to glucose metabolism, reducing ATP and increasing myocardial stress.	Impaired OXPHOS forces reliance on less efficient glycolysis for energy, leading to energy deficits and neuronal dysfunction. Increased lactate and metabolic stress contribute to neurodegeneration.	Both exhibit a shift from OXPHOS to glycolysis under stress, reducing ATP production and energy efficiency	[103,106,124,125]
Electron Transport Chain (ETC)	Dysfunction in complexes I-V reduces ATP, increases ROS, and impaired mitochondrial membrane potential, causing oxidative stress and affecting energy supply for cardiac function. Contributes to heart failure, arrhythmias, and decreased cardiac output.	Dysfunction, particularly in Complex I, impairs NADH oxidation, reducing ATP production, increasing ROS and causing oxidative damage. Contributes to neuronal death and progressive loss of dopamine-producing neurons.	ETC dysfunction reduces ATP production and increases ROS, causing oxidative stress and cellular damage in both conditions.	[106,126,127]
ROS Production	Exacerbated by impaired ETC function and increased electron leakage. Activation of NADPH oxidase and inflammatory pathways further contributes to the heightened ROS levels, promoting oxidative stress and cellular damage in the heart. Oxidative damage to lipids, proteins, and DNA, as well as cellular apoptosis, necrosis and fibrosis.	Increased ROS from mitochondrial dysfunction and dopamine dysregulation. Inflammatory responses triggered by glial activation in response to neuronal damage contribute to ROS production, amplifying oxidative stress.	Increased ROS production causes oxidative stress and cellular damage in both conditions.	[128,129]
Mitochondrial Dynamics	Reduced MFN1/MFN2 and dysregulated DRP1 lead to mitochondrial fragmentation and dysfunction. Reduced mitochondrial connectivity and compromised distribution of mitochondrial components. Impaired mitochondrial quality control, reduced ATP production, increased oxidative stress, contributing to cardiac dysfunction and myocardial damage.	Impaired MFN1/MFN2 and excessive DRP1 activity lead to fragmentation and neuronal death.	Dysregulation in dynamics impairs energy production and increases oxidative stress in both.	[130,131]
Mitochondrial Biogenesis	Decreased PGC-1α and TFAM expression reduce mitochondrial DNA replication and ATP production. Leads to compromised cardiomyocyte energy supply. Mutations or loss of function in PINK1/PRKN.	Disrupted PGC-1α and PARIS signalling impair biogenesis and function. Mutations or loss of function in PINK1/PRKN reduce mitochondrial number and function.	Both involve dysregulation of PGC-1α and PINK1/PRKN, reducing biogenesis and increasing stress.	[132,133,134]
Mitochondrial Ca^2+^ Regulation	Disrupted calcium uptake/efflux through the mitochondrial calcium uniporter (MCU) and sodium-calcium exchanger (NCLX), respectively. Dysregulated ER-mitochondria transfer through voltage-dependent anion channel (VDAC). Leads to opening of mPTP and loss of membrane potential, oxidative stress, impaired metabolism, and cell death.	NCLX inhibition in familial forms of PD with PINK1 deficiency impairs calcium efflux, leading to calcium overload. This triggers the opening of the mPTP. α-synuclein has been suggested to interact with mitochondrial calcium regulatory proteins, disrupting calcium homeostasis.	Dysregulated calcium handling affects mitochondrial function and cell survival in both diseases.	[31,135]
Mitophagy	Impaired PINK1/PRKN pathways reduce degradation of dysfunctional mitochondria. Leads to oxidative stress and compromised cardiac function.	Dysfunction in PINK1/PRKN impairs clearance, leading to neurodegeneration and inflammation. Reduced lysosomal clearance of dysfunctional mitochondria exacerbates energy deficits and promotes accumulation of α-synuclein aggregates. Increased vulnerability to toxins and oxidative stress, triggering neuroinflammation and glial activation.	Dysfunctional mitophagy pathways cause accumulation of damaged mitochondria, increasing stress.	[136,137,138,139]
Lipid Peroxidation (LPO)	Increased oxidative stress and LPO products, leading to impairment of mitochondrial respiration, cardiac ischaemia–reperfusion injury and diabetes.	LPO products by oxidative stress induce the generation, accumulation, and modification of α-synuclein in dopaminergic neurons resulting in mitochondrial dysfunction.	Increased oxidative stress and LPO products, especially related to promoting mitochondrial dysfunction.	[140,141]

OXPHOS, oxidative phosphorylation; ATP, Adenosine triphosphate; ROS, reactive oxygen species; NADH, nicotinamide adenine dinucleotide; ETC, electron transport chain; NADPH, nicotinamide adenine dinucleotide phosphate hydrogen; MFN1/2, Mitofusin-1/2; DRP1, Dynamin-related Protein; PGC-1α, Peroxisome proliferator-activated receptor-gamma coactivator; TFAM, mitochondrial transcription factor A; PINK1, PTEN-induced kinase 1; PRKN, parkin RBR E3 ubiquitin protein ligase; Ca^2+^, calcium ions; mPTP, mitochondrial permeability transition pore; PD, Parkinson’s disease; LPO, lipid peroxidation.

### 3.2. Mitochondrial DNA Defects

Mitochondria possess their own circular, double-stranded DNA, distinct from other cellular organelles [142]. The encoded components in mitochondrial DNA (mtDNA) are essential for cellular energy-generation, and defects in mtDNA can impair mitochondrial function [143]. The impact of mtDNA mutations on mitochondrial energy production deficiency is particularly pronounced in organs and tissues with inherent high energy demands, such as cardiac and neuronal tissues. Within these tissues, mtDNA mutations have the potential to induce myopathy, atrophy and progressive degeneration processes [13]. For instance, mtDNA defects play an important role in the development and progression of myocardial remodelling and failure after myocardial infarction (MI), at least in part, by inducing mitochondrial oxidative stress and damage to mtDNA, which leads to a decrease in mtDNA copy number, number of mitochondrial RNA (mtRNA) transcripts and oxidative capacity [144]. The pathways by which mtDNA mutations impact cardiac function involve a decrease in tRNAs and protein synthesis, including those vital for mitochondrial OXPHOS, leading to an increase in ROS production, subsequent mitochondrial oxidative stress and eventual apoptosis [145].

Similarly, studies have found brain region-specific accumulation of mtDNA damage in PD neuronal culture and animal models, as well as in human post-mortem brain tissue [146]. While mtDNA sequencing did not identify specific pathogenic mutations as a distinctive PD signature, an age-related rise in mtDNA deletions linked to respiratory chain dysfunction was observed in dopaminergic (DA) neurons of the substantial nigra [147]. These deletions were more prevalent in PD patients compared to age-matched controls, particularly in the substantia nigra, indicating a potential association with PD development [147].

Due to the proximity of mtDNA to the electron transport chain within mitochondria, it is more susceptible to damage from free radicals produced during oxidative phosphorylation [142]. The mutation rate of mtDNA is notably higher compared to nuclear DNA, and current knowledge attributes ROS damage as a major driver of mtDNA mutagenesis [145]. Additionally, research suggests that the mitochondrial DNA repair system may be less efficient than that of nuclear DNA, and mtDNA lacks the protective histone proteins, rendering it more susceptible to harmful agents [148]. If left unrepaired, mtDNA can disrupt the ETC, leading to increased ROS production thus initiating a vicious cycle that ultimately results in cellular energy depletion and apoptosis [149].

### 3.3. Genetic Susceptibility

Research on inherited forms of PD has identified several genes linked to mitochondrial dysfunction [29]. While the mitochondrial genetic factors associated with cardiac dysfunction are less extensively documented, the substantial overlap in mitochondrial dysfunction between cardiac pathology and PD suggests a potential shared genetic basis. Leading examples include Parkin RBR E3 Ubiquitin Protein Ligase (PRKN), PTEN-induced kinase 1 (PINK1) and Parkinson disease protein 7 (PARK7), with robust evidence linking them to the development of PD [150]. PRKN and PINK1 gene mutations are recognised as primary causes of autosomal recessive early-onset PD, with PRKN accounting for up to 42.2% of cases with an onset before the age of 20, and PINK1 representing 1–9% of all genetic PD [151,152]. In normal physiology, the shared pathway between PINK1, a protein kinase, and PRKN, an E3 ubiquitin ligase, serves to identify damaged mitochondria and activate a recruitment mechanism for the removal and replacement of dysfunctional components [136,153]. This PINK1/PRKN-mediated mitophagy critically governs cellular fate by eliminating impaired mitochondria, and components of this quality control pathway have already been found mutated in PD or linked to an elevated vulnerability to PD [136,153,154,155,156]. The loss of PRKN has been associated with dopaminergic neuronal loss and the accumulation of damaged mitochondria [157]. Interestingly, mutations in these genes also significantly contribute to the development of cardiac dysfunction [158]. PINK1 absence increases oxidative stress levels and disrupts mitochondrial function in cardiomyocytes, leading to cardiac dysfunction and hypertrophy [159]. PRKN deficiency exacerbates cardiac injury and reduces survival following myocardial infarction [158]. Therefore, targeting the modulation of PINK1/PRKN-mediated mitophagy could be considered a potential therapeutic approach to treat cardiac dysfunction [154].

An additional gene implicated in PD is PARK7, found within the mitochondria which serves as a protective factor against oxidative neuronal damage [142]. Its involvement in PD development has been well-documented [160,161]. Clinically, individuals with PARK7 mutations in PD exhibit an early onset of symptoms like dyskinesia, rigidity and tremors, amongst other PD-like symptoms [162]. In cardiac pathophysiology, PARK7 is associated with cardio-protection through the regulation of cardiac injury in models of I/R and acute myocardial infarctions [163,164,165]. Further investigation into the genetic underpinnings of both conditions will contribute valuable insights for targeted interventions and a more comprehensive understanding of their shared genetic susceptibility.

### 3.4. Mitochondrial Dynamics

Mitochondria exhibit a complex ability to adapt to varying physiological demands through dynamic processes known as fusion and fission. These intricate mechanisms play a role in shaping mitochondrial structure, function and cellular health [166]. Fusion involves the merging of two separate mitochondria into a singular organelle. This process is driven by large guanosine triphosphate (GTP)-hydrolysing enzymes of the dynamin superfamily—Mitofusin 1 (MFN1), MFN2 and Optic atrophy 1 (OPA1). MFN1 and MFN2, positioned on the outer membrane, orchestrate the fusion of outer membranes, while OPA1 is associated with the inner mitochondrial membrane [12]. Mitochondrial fission involves the division of a single mitochondrion into two organelles—a process primarily regulated by the dynamin-related protein 1 (DRP1). Upon activation, DRP1 translocates from the cytosol to the mitochondria. This translocation is facilitated by specific adaptor proteins, such as Fission protein 1 (FIS1), mitochondrial division protein 1 (MDV1) and mitochondrial fission factor (MFF), which are located on the outer mitochondrial membrane. Once at the outer membrane, DRP1 undergoes GTP-dependent oligomerisation, forming a spiral ring around the tubule, ultimately leading to its constriction and division. Dysregulation of fusion and fission processes can lead to distinct mitochondrial network morphologies. These morphological imbalances can influence cellular function and contribute to a range of physiological and pathophysiological conditions [166].

Maintaining proper mitochondrial dynamics is essential for the structural and functional integrity of the heart, both under normal conditions and during periods of stress. Imbalances in fusion or fission processes can result in either elongated or fragmented mitochondria, leading to compromised mitochondrial function (Table 1). In vitro studies suggest that increased mitochondrial fusion promotes cell senescence [167]. Animal models with inhibited mitochondrial fission exhibit adverse effects, exacerbating myocardial fibrosis and cardiomyocyte necrosis [168]. Reduced expression of DRP1 has been associated with heightened mitochondrial depolarisation in the heart, indicating its critical role in maintaining mitochondrial function [169]. In experimental models, mutations affecting DRP1 interactions have resulted in elongated mitochondrial networks, decreased mitochondrial enzyme complexes, ATP depletion and ultimately heart failure [170]. Interestingly, increased mitochondrial fission, predominantly mediated by upregulated DRP1, has been shown to play a significant role in I/R injury, contributing to mitochondrial dysfunction and decreased contractility [171]. Increased DRP1 also participates in opening the mitochondrial permeability transition pore (mPTP), exacerbating heart dysfunction in various pathological conditions such as I/R injury and chronic β-adrenergic receptor activation [172]. Evidence also suggests that DRP1-mediated mitochondrial fission contributes to cardiomyocyte death in conditions such as pressure overload and myocardial infarction [169]. Inhibiting DRP1, either genetically or pharmacologically, has shown promise in preventing mPTP opening, reducing infarct size and improving cardiovascular function in animal models of acute myocardial infarction [169]. Moreover, studies inducing heart failure in animal models and treating them with DRP1 inhibitors have demonstrated improvement in left ventricular function, accompanied by reduced expression of autophagy markers [170].

In PD, reduced DRP1 activity in mitochondria of PD mouse models with α-synucleinopathy lead to enlarged neuronal mitochondria and compromised neuronal integrity [173]. Deletion of DRP1 in mouse dopaminergic neurons prompts rapid degeneration of dopamine terminals and cell bodies in the midbrain by impairing mitochondrial dynamics in axons, resulting in reduced mitochondrial mass and disrupted mitochondrial movement coordination [174]. Interestingly, other studies suggest that neurotoxins associated with PD trigger DRP1-mediated fragmentation of mitochondria, indicating a predominant role for mitochondrial fission in pathology [175]. Exposure of cultured rat cortical neurons to acute rotenone induces rapid mitochondrial fragmentation and cell death, dependent on DRP1 [29]. The pharmacological inhibition of DRP1 by mitochondrial division inhibitor (Mdivi-1) in PD rats not only significantly alleviates behavioural deficits but also modulates mitochondrial function, enhances biogenesis and boosts the production of new-born dopaminergic neurons in the substantia nigra pars compacta [176].

The involvement of fusion proteins in mitochondrial dynamics in the heart has also been explored. Dysregulation of fusion proteins like MARF (analogous to human MFN) and OPA1 in Drosophila and mice models has been linked to cardiomyopathy and impaired heart function, whereas the overexpression of human MFN1/2 has shown promise in mitigating these effects [177]. In aging hearts, imbalanced mitochondrial dynamics contribute to cardiac dysfunction, characterised by reduced MFN1/2 and increased OPA1 and DRP1 expression [177]. Ischemic heart failure correlates with decreased OPA1 expression, exacerbating apoptosis and heart failure progression [178]. Studies involving specific deletion of MFN2 in cardiomyocytes demonstrate impaired mitochondrial fusion and cardiac hypertrophy, and altered expressions of MFN1/2 have been noted in ischemic and non-ischemic heart failure [179,180]. Inhibition of fission has shown efficacy in reducing I/R injury [181]. OPA1 mutant heart tissue displays increased ROS and compromised mitochondrial function, suggesting a potential role in heart disease progression [181].

In PD, deletion of MFN2 induces aberrant mitochondrial morphologies and impairs ETC activity, leading to a severe loss of nerve terminals in the striatum [130]. Additionally, mutations in the OPA1 gene have been associated with inherited forms of PD and other dementia, potentially through destabilisation of the cristae structure and activation of the apoptotic pathway [182]. MFN2 ablation also disrupts PRKN signalling initiating aberrant mitophagy [183]. Furthermore, overexpression of α-synuclein in transgenic mice results in reduced MFN1/2 protein levels, corresponding to diminished mitochondrial fusion and smaller mitochondria, while α-synuclein knockdown induces mitochondrial elongation [184].

The precise mechanisms governing fusion/fission protein modulation varies across different disease contexts, and further research is essential for developing effective interventions to mitigate disease progression. Promisingly, DRP1 inhibitors such as Mdivi-1, have shown potential in restoring the balance between fusion and fission dynamics [33,185]. Additionally, flavonoids have emerged as a therapeutic avenue for modulating mitochondrial fission. Administration in PD mouse models has demonstrated the restoration of mitochondrial dynamics by downregulating DRP1 expression and upregulating MFN1 expression in the hippocampus [186]. In cardiac pathology, interventions like 7,8-dihydroxyflavone (7,8-DHF) treatment in myocardial ischemic mice have shown promise in ameliorating cardiac dysfunction and cardiomyocyte abnormalities by suppressing mitochondrial fission [187]. The selective peptide inhibitor P110, targeting excessive mitochondrial fission, also emerges as a promising pharmacological intervention [188]. P110 has demonstrated cardioprotective effects by preserving cardiac mitochondria from I/R injury-induced damage, reducing myocardial infarct size and improving cardiac function in CVD models [170]. Similarly, in PD models, P110 shows neuroprotective properties by preserving mitochondrial function, mitigating neuronal death and potentially slowing neurodegeneration [189]. With its ability to safeguard mitochondria from damage and enhance mitochondrial function, these approaches hold promise for further investigation in clinical trials as therapeutic strategies for both cardiac dysfunction and PD.

### 3.5. Biogenesis Deficits

Mitochondrial biogenesis is a cellular response to heightened energy demands triggered by developmental signals and environmental stressors, involving the generation of new mitochondria from existing ones [190]. Mitochondria are descendants of an alpha-protobacteria endosymbiont and possess their own genetic material and capability for self-replication [191]. This process is vital for maintaining cellular balance, coordinating the equilibrium between generating new mitochondria through biogenesis and removing damaged ones via mitophagy [190]. Several growth factors are involved. Peroxisome proliferator-activated receptor-gamma coactivator (PGC-1α) serves as a central regulator, tiggering transcription factors like nuclear respiratory factors-1 (NRF1) and NRF2. These initiate the transcription of nuclear-encoded respiratory chain proteins expressed by the nuclear genome, ultimately leading in mitochondrial DNA replication [192,193]. Other transcription factors such as mitochondrial transcription factor A (TFAM) and dimethyladenosine transferase 2 (TFB2M) can then act in response to intracellular oxidative stress [192]. PGC-1α, regulated by AMP-activated protein kinase (AMPK), is often reduced in many diseases (Table 1) [193].

Mitochondrial biogenesis plays a crucial role in cardiac function [116]. In the heart, this association stems from diminished mitochondrial function in cardiac and skeletal muscles, characterised by changes in mitochondrial proteins and reduced levels of PGC-1α, along with its downstream transcription factors NRFs and TFAM [194]. This reduction in cardiac PGC-1α is consistently observed in various heart failure models [116,195,196,197]. Hearts with deletions in PGC-1α or PGC-1β are more susceptible to heart failure when exposed to pressure overload [196]. The chronic deactivation of the PGC-1α axis leads to decreased ATP production, impacting energy transfer and utilisation, ultimately resulting in decreased energy efficiency [116,194,195]. Research on PGC-1α knockout and transgenic mice have highlighted its influence on various pathways beyond mitochondrial biogenesis, underscoring the critical role of PGC-1α expression in determining energetic states [198]. Regarding mitochondrial biogenesis in PD, recent research involving PRKN-deficient human dopaminergic neurons found that the main driver of mitochondrial dysfunction was defective biogenesis [4,33]. This was attributed to increased levels of the PRKN substrates PARIS, leading to decreased PGC-1α expression [4,33]. Studies on PGC-1α knockout mice demonstrated a reduction in the expression of genes responsible for mitochondrial respiration, resulting in decreased enzymatic activities and lower ATP concentrations [199]. Additionally, diminished expression of a specific set of 425 PGC-1α responsible genes is closely correlated with PD pathology and subclinical Lewy body neuropathology. PGC-1α activation boosts expression of nuclear-encoded mitochondrial respiratory chain subunits, safeguarding dopaminergic neurons from damage by mutant α-synuclein or rotenone [199].

The reduction of the PGC-1α axis during cardiac dysfunction and PD, along with its ability to enhance energy production and reduce oxidative stress, highlights its potential as a target for preventing and treating these diseases [195,199]. One approach focuses on reducing acetylation of PGC-1α and PGC-1β proteins, negatively regulating their activity [195]. SIRT1, a deacetylase for PGC-1α/β, can be activated by compounds like resveratrol, leading to enhanced PGC-1 activity [200,201]. Resveratrol exhibits cardioprotective effects in various heart failure models through SIRT1 activation [202,203]. It also plays a neuroprotective role through the SIRT1/PGC-1α pathway in PD models [204,205].

Exercise has also emerged as a therapeutic strategy, promoting mitochondrial biogenesis and function via the SIRT1/PGC-1α pathway [33,198]. In a unilateral PD rat model, one week of intermittent moderate treadmill exercise prevented a decrease in PGC-1α and NRF1 expression [33]. Exercise training induced activation of the SIRT1 pathway, reducing cardiomyocyte apoptosis and oxidative damage [206]. Increased levels of SIRT and PGC-1α were found after moderate long-term exercise in rats and mice, suggesting that exercise can upregulate the SIRT1/PGC-1α pathway. This evidence supports the idea that exercise-induced SIRT1/PGC-1α activation promotes mitochondrial biogenesis and neuronal health [201,207]. This mechanism may contribute to the neuroprotective and cardioprotective benefits observes with exercise.

### 3.6. Calcium Dysregulation

Calcium ions (Ca^2+^) serve as versatile signalling molecules in various physiological processes, encompassing muscle contraction, neuronal excitability, cell migration and cell growth [208]. Mitochondria play a pivotal role in regulating cellular Ca^2+^. Beyond this role, mitochondrial Ca^2+^ has additional functions, such as influencing ATP production, cytosolic calcium levels and cell fate, including apoptosis control [208]. Extensive evidence underscores the critical role of mitochondrial Ca^2+^ in ATP production. In the context of the heart, the influx of Ca^2+^ into mitochondria assumes a critical stance in sustaining ATP production, thereby underpinning the contractile activity of cardiomyocytes [209]. Similarly, in neurons, mitochondrial Ca^2+^ buffering is essential for shaping Ca^2+^ signals and regulating various Ca^2+^-dependent functions, such as neuronal excitability, synaptic transmission, gene expression and neuronal toxicity [81]. Any disruption in tightly regulated mitochondrial Ca^2+^ levels initiate a cascade of molecular malfunctions, resulting in mitochondrial dysfunction and abnormal Ca^2+^ signalling, leading to cellular degeneration.

Insufficient levels of Ca^2+^ within cardiac mitochondria can compromise their functionality, diminishing energy production and potentially inducing cellular damage or death (Table 1) [209]. Conversely, excessive Ca^2+^ accumulation can also severely disrupt cellular functions [210]. In heart failure, there is an imbalance between Ca^2+^ and ATP, leading to the oxidation of pyridine nucleotides within the mitochondrial matrix. This process may diminish ATP synthesis and cardiomyocyte contraction, while also triggering oxidative damage through NADPH oxidation, subsequently contributing to maladaptive cardiac remodelling [135]. Research findings indicate a substantial elevation in mitochondrial Ca^2+^ and ROS levels in a murine model of heart failure post-myocardial infarction [211]. Leaky ryanodine receptors (RyR2) channels on the sarcoplasmic reticulum (SR), which cause SR Ca^2+^ leakage, leads to mitochondrial Ca^2+^ overload and mitochondrial dysfunction. Mitigating RyR2 oxidative modification or phosphorylation has demonstrated a reduction in SR Ca^2+^ leak, leading to improved mitochondrial function and decreased severity of heart failure [211]. In instances of I/R injury, mitochondrial Ca^2+^ overload induces a sudden rise in mitochondrial membrane permeability, a significant driver of cell death [210]. Studies involving genetic deletion of the mitochondrial calcium uniporter (MCU) in adult mouse cardiomyocytes have shown protective effects against I/R injury. This intervention reduces mitochondrial Ca^2+^ overload, diminishes activation of the mitochondrial permeability transition pore, decreases infarct size and enhances cardiac function post-I/R [212]. Other studies, however, indicate that while MCU deletion leads to less mitochondrial Ca^2+^ accumulation during ischemia, it does not entirely prevent it, suggesting that mitochondrial Ca^2+^ overload during ischemia is not exclusively dependent on MCU [213]. Overall, the maintenance of a balanced level of mitochondrial Ca^2+^ is crucial for cardiomyocyte survival and function [209].

Chronic elevated Ca^2+^ levels, resulting from altered Ca^2+^ transient handling, are also a significant pathological hallmark of PD. This Ca^2+^ dysregulation affects cellular signalling and damages mitochondria, leading to cell death [31]. In PD neurons, mitochondrial Na^+^/Ca^2+^ exchange (mNCX) mechanisms play a crucial role in neuronal Ca^2+^ homeostasis, acting as safeguards against cell death from excessive mitochondrial Ca^2+^ accumulation [214,215]. Recent findings suggest that the mitochondrial kinase PINK1 regulates calcium efflux from mitochondria through mNCX [214,216]. PINK1 deficiency leads to mitochondrial calcium overload, triggering increased ROS production, inhibiting glucose transport, reducing substrate delivery and compromising function [216]. Activating mNCX in PINK1 deficient models rescues pathogenic mitochondrial Ca^2+^ efflux, mitochondrial depolarisation and neuronal cell death through the protein kinase A (PKA) pathway [217]. In some cases, mitochondrial Ca^2+^ overload is linked to prolonged cytosolic Ca^2+^ levels [218]. The activation of plasma membrane L-type Cav1.3 Ca^2+^ channels induce independent, rhythmic pace making in dopaminergic neurons. Aging and stress factors associated with PD further intensify this neuronal activity [218]. Additionally, PD dopaminergic neurons exhibit a restricted Ca^2+^ buffering system. These elements collectively contribute to heightened mitochondrial Ca^2+^ uptake and activation of OXPHOS. While ensuring practical ATP synthesis, this process may result in mitochondrial hyperpolarisation, the generation of ROS, mitochondrial damage and the formation of defective organelles [218].

### 3.7. Mitophagy Impairment

Mitophagy is a highly coordinated cellular process involving the identification, capture and transport of damaged mitochondria to lysosomes for degradation [219]. This mechanism comprises three main phases: recognition of damaged mitochondria, formation of autophagic membranes around these targeted mitochondria and subsequent fusion of these autophagosomes with lysosomes [192]. This process is intricately linked with mitochondrial fusion, fission and biogenesis, ensuring the maintenance of appropriate mitochondria morphology, quantity, quality and turnover [220]. Regulation of mitophagy involves a network of proteins, including those associated with mitochondrial fusion (MFN1, MFN2, OPA1) and fission (DRP1, FIS1), as well as B-cell lymphoma 2 (BCL-2) family proteins and the PINK1/PRKN pathway [221]. Mitophagy plays a pivotal role in responding to various forms of stress, and dysregulation of this process contributes to the onset of several diseases (Table 1) [222,223].

In cardiac pathology, the functional significance of cardiac mitophagy has been revealed by loss-of-function mouse models of autophagy [224]. Mice with cardiac-specific autophagy related 5 (ATG5) or autophagy related 7 (ATG7) ablation, required for optimal autophagic responses, develop cardiac hypertrophy, left ventricular dilation, contractile dysfunction and premature death, along with mitochondrial misalignment and aggregation [225]. Biopsies from individuals with heart failure show downregulated autophagy-specific genes (beclin1 and LC3-II), indicating a potential association between mitophagy and heart failure development. Subsequent experiments on heart failure models have revealed that insufficient mitophagy aggravates heart injury [137]. The impact of PINK1 and PRKN is highly responsible for mitophagy impairment, and this impact extends to the cardiac system [226]. In Drosophila models, genetic deletion of either PINK1 or PRKN results in mitochondrial dysfunction and compromised cardiac contractibility [224]. Patients with end-stage heart failure exhibit reduced expression of PINK1, and its loss leads to mitochondrial dysfunction and excessive ROS accumulation [154]. Mutations leading to the reduced function of the PINK1/PRKN pathway have also been associated with the development of PD [152,227]. The expression of several mitophagy-related genes is altered in DA neurons derived from PRKN knockout isogenic induced pluripotent stem cells (iPSCs) [228]. Neurons differentiated from iPSCs carrying PRKN or PINK1 mutations demonstrate aberrant accumulation of phospho-ubiquitin and aggregated mitochondria [228]. Moreover, PINK1 and PRKN null Drosophila exhibit learning and memory abnormalities, weakened circadian rhythms, and underlying electrophysiological irregularities in clock neurons [136]. Overall, the evidence indicates mitophagy to play a role in the degeneration of dopaminergic neurons observed in PD progression [229].

The exploration of nutritional and pharmacological interventions to modulate autophagy/mitophagy as potential therapeutic strategies holds promise. Inhibitors of mTOR have demonstrated the ability to induce autophagy, offering protection against various cardiac pathologies and extending lifespan across different species [230]. Notably, the mTOR inhibitor rapamycin has been identified as contributing to the safeguarding of dopaminergic neurons and enhancing behavioural outcomes in mice with PD [230]. While no specific study has focused on the impact of mTOR inhibitors in targeting mitophagy for the treatment of cardiac dysfunction, research has shown the cardioprotective effects of rapamycin in pressure-overloaded and ischemic heart diseases [231]. AMPK activators have also emerged as potential therapeutic strategies. AMPK serves as a cellular energy sensor regulating metabolic pathways, including autophagy and mitophagy. Activation of AMPK promotes mitophagy and enhances mitochondrial function [232]. Pharmacological agents like metformin and resveratrol, known for activating AMPK, hold potential therapeutic benefits in cardiac function and PD [233,234]. Metformin, commonly used in type 2 diabetes (T2D) management, exerts neuroprotective effects through AMPK activation, reducing mitochondrial dysfunction, oxidative stress and α-synuclein aggregation [235,236]. Moreover, metformin has been shown to exert beneficial effects in myocardial ischemic injury, diabetic cardiomyopathy, cardiotoxicity and ventricular dysfunction [237]. Another therapeutic avenue is the use of nicotinamide adenine dinucleotide (NAD+) precursors. NAD+ plays a crucial role in cellular energy metabolism and mitochondrial function. Its levels decline with age and in various disease conditions, impairing mitochondrial health and contributing to disease progression. NAD+ precursors such as nicotinamide riboside (NR) and nicotinamide mononucleotide (NMN) have been shown to enhance mitophagy and improve mitochondrial function, offering potential therapeutic benefits for both cardiac dysfunction and PD [238,239]. For instance, NR was shown to prevent neuronal cell loss in patient-derived cell lines and Drosophila models of PD [240]. Supplementation with NAD+ precursors has been linked to significant reduction in systolic and diastolic blood pressure as well as C-reactive protein concentrations, indicating a decrease in inflammation and suggesting potential benefits for cardiovascular risk factors [241]. In heart failure, NMN administration mitigates mitochondrial dysfunction by reducing mitochondrial protein hyperacetylation, preserving mitochondrial ultrastructure, reducing ROS, preventing cell death and enhancing long-chain fatty acid oxidation [242]. These findings suggest a promising avenue for targeting mitochondrial health in the treatment of both cardiac comorbidities and PD.

### 3.8. Oxidative Stress and Lipid Peroxidation

Recently, growing evidence has highlighted oxidative stress and associated mitochondrial dysfunction to be critical players in the pathophysiologic processes involved in neurodegenerative conditions, suggesting a new paradigm for therapeutic treatments. Oxidative stress is characterized by an overproduction of ROS within cells. It results from perturbations in homeostatic mechanisms involved in pro- and antioxidant balance, whereby the balance of ROS and antioxidants observed under physiological conditions is disrupted due to the overproduction of free radicals. The most common ROS include singlet oxygen (O_2_), superoxide anion radicals (O_2_^•−^), hydroxyl radicals (HO^•^) and hydrogen peroxide (H_2_O_2_) [243]. These excess free radicals can lead to not only protein deposition and DNA damage, but also degradation of proteins and lipids [244].

The mitochondrion is the single most important cellular organelle involved in the generation of ROS [245], primarily as a consequence of aerobic respiratory processes, which are mediated by protein complexes within the inner mitochondrial membrane. Notably, the inner mitochondrial membrane is prone to lipid peroxidation (LPO) [246]. LPO is the formation of a free radical chain as a result of damage to the phospholipid layer of the cell through oxygen derived-free radicals [247]. Indeed, oxidative damage of lipids results in the formation of LPO products, which further attack lipids, resulting in the release of lipid peroxyl radicals, hydroperoxides, which subsequently target and induce the breakdown of proteins and DNA.

Lipids are major components of the structural and functional organization of the central nerve system (CNS), comprising almost 60% of the dry mass of human brain. Lipid properties and the effects in the CNS are directly determined by the proportion of specific fatty acids within their molecular structure and, in particular, by the content of long-chain polyunsaturated fatty acids (PUFAs) [248,249]. PUFAs can be classified as omega-3 (n-3) and omega-6 (n-6) fatty acids. Interestingly, within healthy dopaminergic neurons, LPO products induce the generation and accumulation of misfolded α-synuclein, resulting in insufficient dopamine production and the development of PD [140]. It has been recently demonstrated that reduced levels of glutathione, an antioxidant critical for protecting dopaminergic neurons in the SNpc from free radical damage, directly increases LPO levels [250]. Furthermore, clinical studies have reported significant upregulation of acrolein levels in the brain with PD [140,251]. Acrolein, which is an α, β-unsaturated aldehyde produced by the lipid peroxidation of PUFAs, promotes the initiation of LPO and further elevation of oxidative stress, as indicated by acrolein-induced increases in 4-hydroxy-2-alkenal (HNE) levels, formed by the peroxidation of omega-6 (n-6) PUFAs [252]. Additionally, acrolein acts on the modification of α-synuclein in dopaminergic neurons, leading to mitochondrial dysfunction and resulting in the ROS-mediated apoptosis of affected neurons [253]. Importantly, elevated levels of HNE and acrolein have both been reported in the CSF of living PD patients [253,254,255].

Numerous studies have also found that there are functional consequences in the heart following exposure to acrolein [256,257,258]. Due to the established role of LPO products in heart failure and cardiac ischaemia–reperfusion injury [259] and diabetes [260], they may play an underappreciated role in the pathophysiology of these disease processes in PD patients and warrant further investigation [257,261,262].

## 4. Future Perspectives

While there is a strong association between Parkinson’s disease (PD) and cardiac complications, establishing a definitive cause-and-effect relationship remains challenging. Individuals with PD often exhibit a heightened susceptibility to cardiac dysfunction, including increased risk of myocardial infarction, heart failure and overall mortality. This review explores potential commonalities in the molecular mechanisms underlying cardiac comorbidities in PD. In this respect, it would be worthwhile to investigate whether this association is bidirectional—that is, whether there is also an increased risk of neurodegenerative diseases associated with cardiac conditions. The existing literature on this topic is sparse, indicating a valuable area for future research. Investigating these associations could provide important insights into the broader implications of the connection between neurodegenerative diseases and cardiac health. For instance, emerging evidence suggests that chronic cardiac dysfunction, such as heart failure, may raise the risk of developing neurodegenerative diseases like Alzheimer’s disease (AD) and Parkinson’s disease (PD) [263,264,265]. Age-related changes in cardiac haemodynamics, including alterations in cardiac output and arterial stiffness, could impair brain health and accelerate cognitive decline by affecting brain structure and function, potentially interacting with neurodegenerative processes in aging adults [266]. Cardiac dysfunction may also lead to reduced cerebral perfusion, which can impair cognitive function and accelerate neurodegeneration [265]. Additionally, cardiac issues may exacerbate existing neurodegenerative conditions by inducing systemic inflammation, further compromising brain health [267]. Interestingly, a recent study found that while myocardial infarction (MI) was associated with a higher risk of vascular dementia, particularly in patients who experienced a stroke post-MI, it was not linked to an increased risk of Alzheimer’s disease or other dementias [268]. Furthermore, another study revealed that survivors of MI had a reduced risk of developing Parkinson’s disease and secondary parkinsonism, indicating a possible inverse relationship between cardiovascular risk factors and these conditions [269]. These findings highlight the need for further research to clarify the pathogenetic and clinical connections between cardiovascular disease and Parkinson’s disease. Such research could inform the development of new therapeutic strategies and improve our understanding of the complex interactions between these conditions.

Understanding the broader links between PD and cardiac comorbidities is crucial. Exploring specific key molecules in PD that may lead to cardiac insufficiency can yield valuable insights into these connections. Notably, few studies in PD patients have focused on identifying the molecular keys responsible for cardiac dysfunction. Several PD-associated genes, such as PRKN, PINK1, PARK7, LRRK2, and α-synuclein, are expressed in cardiac tissue. Although definitive clinical links between these genes and cardiac damage in PD are lacking, preclinical models, as discussed throughout the review, suggest that these genes may exert protective effects on the heart. PINK1 is crucial for heart function, regulating mitochondrial health during stress. Its deficiency leads to cardiac hypertrophy and increased susceptibility to ischemic injury in animal models [270]. PARK7 protects the heart against oxidative stress, with its absence heightening the risk of cardiac damage during ischemia [163]. Mutations in LRRK2 have also been associated with altered heart rate variability in PD patients [271]. Interestingly, aberrant α-synuclein aggregates have been found in the epicardial tissue of undiagnosed PD patients, suggesting a potential link to the disease’s prodromal stage [8,272]. Furthermore, Lewy bodies have been identified in the myocardium of PD patients, particularly in the nerve fibres of arteries and atrial ganglia, while α-synuclein accumulation in myocardial tissue and coronary arteries suggests a possible connection to cardiac sympathetic dysfunction [8,273,274]. Given the elevated risk of heart disease in PD patients, assessing mutations in PD-related genes for potential links to cardiovascular disease is critical. However, despite these associations, direct evidence identifying specific molecular connections between PD and cardiac insufficiency remains limited. Given the overlap of mitochondrial dysfunction in both neurodegenerative and cardiovascular diseases, further research is essential to determine whether these identified molecules are key specific contributors to cardiac comorbidities in PD.

## 5. Conclusions

Mitochondria, crucial for both heart and brain function, are implicated in the development of cardiac dysfunction and PD. Mitochondrial dysfunction in these conditions can manifest through various mechanisms, such as bioenergetic deficits, mitochondrial DNA defects, genetic predispositions, disruptions in mitochondrial dynamics, biogenesis deficits, calcium dysregulation, impaired mitophagy and oxidative stress. Despite this observed association, the precise underlying mechanisms are not entirely clear. Hence, our review aims to explore the potential role of mitochondrial dysfunction as a mediator of cardiac comorbidities in PD. By uncovering common pathways and molecular targets relevant to both cardiac dysfunction and PD, there is potential for the elucidation of shared therapeutic targets. Exploring the role of mitochondrial dysfunction as a potential mechanism linking both conditions can provide new perspectives into their interconnectedness and highlight promising areas for future research.
